# Comparative and phylogenetic analysis based on chloroplast genome of *Heteroplexis* (Compositae), a protected rare genus

**DOI:** 10.1186/s12870-022-04000-1

**Published:** 2022-12-22

**Authors:** Na Duan, Lili Deng, Ying Zhang, YanCai Shi, Bingbing Liu

**Affiliations:** 1 Department of Life Sciences, Changzhi University, 046011 Changzhi, Shanxi China; 2 Institute of Loess Plateau, Shanxi University, 030006 Taiyuan, Shanxi China; 3 Guangxi Institute of Botany, Guangxi Zhuang Autonomous Region and Chinese Academy of Sciences, 541006 Guilin, Guangxi China

**Keywords:** *Heteroplexis*, Chloroplast genome, Phylogenetic analysis

## Abstract

**Background:**

*Heteroplexis* Chang is an endangered genus endemic to China with important ecological and medicinal value. However, due to the lack of genetic data, our conservation strategies have repeatedly been delayed by controversial phylogenetic (molecular) relationships within the genera. In this study, we reported three new *Heteroplexis* chloroplast (cp.) genomes (*H. vernonioides*, *H. impressinervia* and *H. microcephala*) to clarify phylogenetic relationships between species allocated in this genus and other related Compositae.

**Results:**

All three new cp. genomes were highly conserved, showing the classic four regions. Size ranged from 152,984 − 153,221 bp and contained 130 genes (85 protein-coding genes, 37 tRNA, eight rRNA) and two pseudogenes. By comparative genomic and phylogenetic analyses, we found a large-scale inversion of the entire large single-copy (LSC) region in *H. vernonioides*, *H. impressinervia* and *H. microcephala*, being experimentally verified by PCR. The inverted repeat (IR) regions showed high similarity within the five *Heteroplexis* plastomes, showing small-size contractions. Phylogenetic analyses did not support the monophyly of *Heteroplexis* genus, whereas clustered the five species within two differentiated clades within *Aster* genus. These phylogenetic analyses suggested that the five *Heteroplexis* species might be subsumed into the *Aster* genus.

**Conclusion:**

Our results enrich the data on the cp. genomes of the genus *Heteroplexis,* providing valuable genetic resources for future studies on the taxonomy, phylogeny, and evolution of *Aster* genus.

**Supplementary Information:**

The online version contains supplementary material available at 10.1186/s12870-022-04000-1.

## Background

*Heteroplexis* Chang is an oligotypic genus endemic to China, which belongs to the tribe Astereae within the Asteraceae family. It was first described as a monotypic genus (*Heteroplexis vernonioide*), only found in the Longzhou region, Guangxi, China (Chang, 1937). In a recent infrageneric classification of *Heteroplexis*, five species (*H. vernonioide*, *H. microcephala*, *H. sericophylla*, *H. incana* and *H. impressinervia*) were recognized on the number of hermaphroditic flower and leaf characteristics, such as glandular spots, villi, and vein [1, 2]. These genus species occur mainly in limestone areas along the valleys and mountaintops in the Guangxi Zhuang Autonomous Region of southern China. Most *Heteroplexis* species have a very narrow and disjunctive distribution area [3]. Such as, *H. microcephala* has been only recorded in four different towns in Yangshuo county (Guangxi), and *H. incana* has been only found in Liuzhou (Guangxi) [3]. In addition, the main effective pollinators of the *Heteroplexis* species are insects such as hoverflies and *Vespa ducalis*, which are highly susceptible to environmental and climatic factors [4]. As a result, these *Heteroplexis* species are threatened. Most of them have been listed on the National List of Rare and Endangered Plants in China [5]. Conservation strategies are delayed due to a lack of understanding of the interspecific relationships within this genus.

Phylogenetic studies of *Heteroplexis* have been underway since it was first discovered. As early as 1937, when Chang discovered *Heteroplexis vernonioide* for the first time, he thought that its morphological structure was similar to *Brachyactis* (Chang, 1937). Zhang and Kare further attributed the *Heteroplexis* to *Erigeron* - *Conyza* group because ‘it has more female outer florets than hermaphroditic central florets’ [6]. Later on, Shi et al. (2015), using inter simple sequence repeat (ISSR) makers, found that *Heteroplexis* species split into three clades *H. impressinervia* and *H. microcephala* clade, *H. vernonioides* and *H. incana* clade, and *H. sericophylla* clade [3]. In contrast, Hu (2015), using internal transcribed spacer (ITS) and Expressed sequence Tags (ETS) molecular markers, showed that the *Heteroplexis* specimens were located in the tribe Astereae, with *H. sericophylla* and *H. vernonioides* as sister species, and *H. microcephala* close to *Aster* species [7].

In higher plants, the chloroplast (cp.) is a semi-autonomous replication organelle with its own genetic material - the cp. genome [8–10]. The cp. genomes of angiosperms range from 120 to 160 kb in length and exhibit a typical quadripartite circular DNA molecule, which consists of one large single-copy (LSC) region, one small single-copy (SSC) region, and two inverted repeats (IR) [5, 11, 12]. Due to its uniparental inheritance and low recombination and substitution rate, the cp. genome (in whole or as concatenated protein-coding genes fragment) has been used for phylogenetic studies over the past decades, replacing ISSR markers [10, 13]. For example, Kim et al. (1995) constructed a phylogenetic tree of 89 species from Compositae based on chloroplast *ndh*F genes, identifying five major clades [14]. Whereas Panero et al. (2008) further discussed the origin, evolution, and phylogenetic relationships of Compositae using ten chloroplast-encoded genes [15]. With the advent of next-generation sequencing technology, it is more efficient and inexpensive to obtain cp. genome sequences, which generally provide more genetic information than a few gene fragments to solve the phylogenetic relationship of complicated lineages [16–18]. So far, two *Heteroplexis* species (*H. sericophylla* and *H. incana*) cp. genomes have been reported, and phylogenetic trees based on whole cp. genomes showed that *Heteroplexis* is more closely related to *Aster* [5, 19].

In this study, we sequenced and assembled the other three *Heteroplexis* species whole cp. genomes and performed comprehensive structural, sequence, and phylogenetic analyses with previously published *Heteroplexis* spp. cp. genomes. The main goals of this study were: (1) provide new cp. genomes data and perform a comparative genomic analysis among *Heteroplexis* species; (2) elucidate the phylogenetic relationships of *Heteroplexis* species within the genus and family; and (3) identify hypervariable loci for future development of molecular markers to ascertain *Heteroplexis* species identification for conservation and phylogenetic studies.

## Results

### General CP features

Illumina HiSeq paired-end (150 pb) reads were assembled for each *Heteroplexis* species with NOVOPlasty software, obtaining three new full cp. genomes deposited at NCBI (*H. impressinervia*, MN367917; *H. microcephala*, MW795355; and *H. vernonioides*, MN462631), that were compared with the previously published two plastomes of *Heteroplexis* (*H. incana*, MN172194 and *H. sericophylla*, MK942054).

**Fig. 1:**
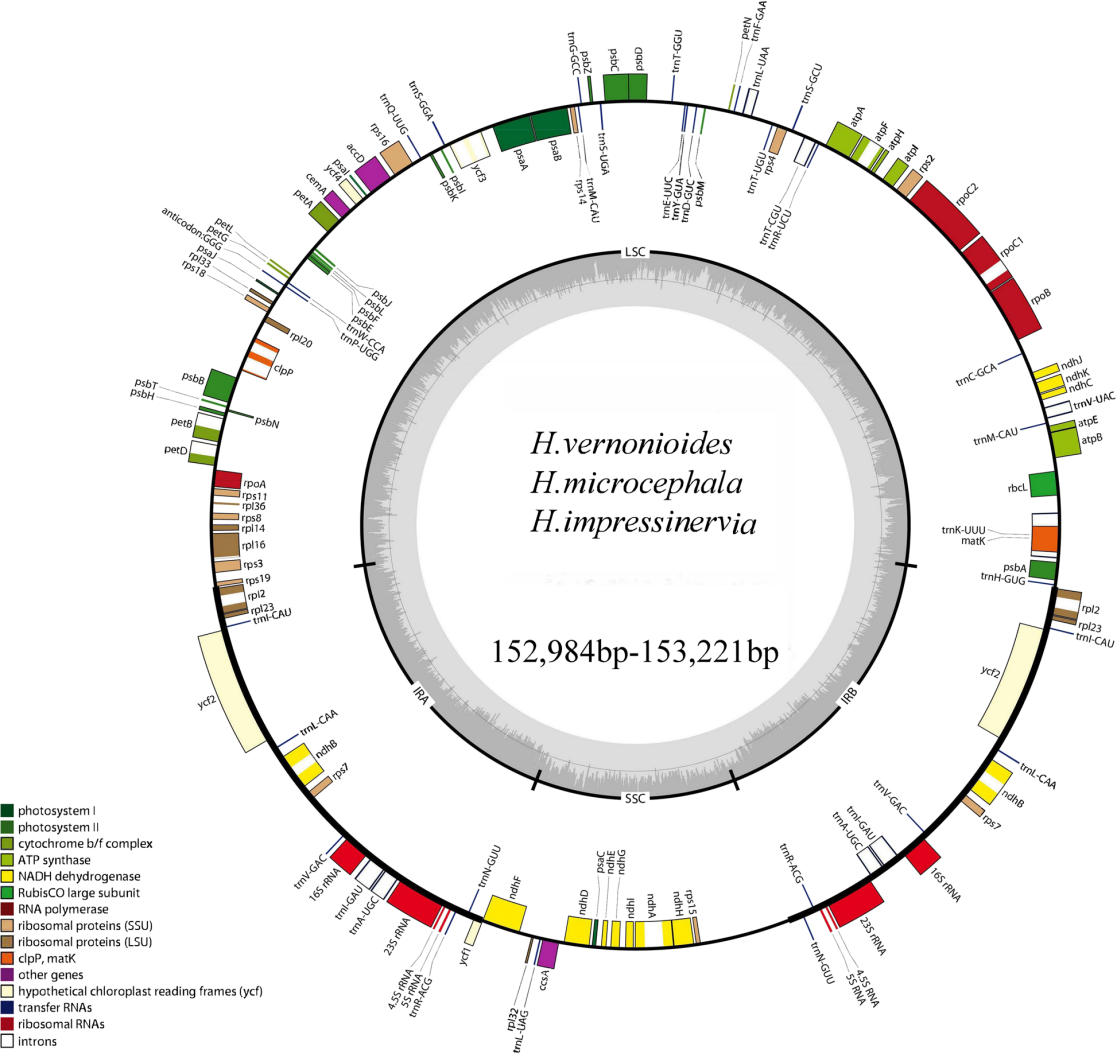
Physical maps of three newly sequenced *Heteroplexis* chloroplast genomes

All five *Heteroplexis* plastomes exhibit a typical quadripartite structure (LSC-IR-SSC-IR), with a conserved gene content, relative gene position and orientation (Fig. 1; Supplementary Table S1). The cp. genome length ranged from 152,605 bp (*H. incana*) to 153,221 bp (*H. vernonioides*), with an average GC content of 37.3%, except for *H. vernonioides* that was 37.2%. The total number of annotated genes in the characterized *Heteroplexis* plastomes was of 132, distributed in 85 (79 + 6) protein-coding genes (PCGs), 37 (30 + 7) tRNAs, and 8 (4 + 4) rRNA genes, and two pseudogenes (*rps*19^φ^ and *ycf*1^φ^). Six tRNA genes and 12 PCGs contained introns, of which 15 (*atp*F, *ndh*A, *ndh*B, *rps*16, *rpoC*1, *pet*B, *pet*D, *rpl*16, *rpl*2, *trn*A-UGC, *trn*I-GAU, *trn*G-UCC, *trn*K-UUU, *trn*L-UAA, and *trn*V-UAC) contained only one intron and three (*clp*P, *rps*12 and *ycf*3) contained two introns (Table S2).

### Repeated sequences

A total of 339 complex repeats sequences, consisting of 227 interspersed repeats (101 forward, 20 reverse, 100 palindrome, and six complementary) and 112 tandem repeats, were identified within plastome genomes using MISA, REPuter and Tandem Repeat Finder software, as described in the Material and Methods section (Figure S1A). For interspersed repeats, the sequence length is mainly concentrated in 30–60 bp, regardless of the forward or palindrome repeats. As for the tandem repeats, most were in the range of 16–40 bp, and there was a repeat over 117 bp in *H. vernonioides.* It is located between the gene *trn*T-UGU and *trn*G-UCC. These tandem repeats were mainly distributed in the non-coding LSC and SSC regions.

Microsatellites are small repeating units (1–6 nucleotide) within a genome nucleotide sequence [20]. The high rate of polymorphism in repeat sequences at the species level makes them one of the most common molecular markers in phylogenetic and population genetics studies [21]. The total number of SSRs range from 85 to 93 (Figure S1B), the majority of which were mononucleotide repeats (34%, with A/T showing the highest numbers), followed by trinucleotide (24%) and dinucleotides (18%), whereas other SSRS types have lower proportions (Figs. S1C, S1D). Mononucleotide repeats may play a more important role in genetic variation than other SSRs types. Our findings are similar to other studies that show that A/T repeats were the most abundant [22]. In addition, the analysis of SSR locations revealed that most SSRs were distributed in the non-coding, intergenic, and intron regions. These SSRs can be used to develop specific markers, which can be key in studying systematics and evolution of the family, with conservation purposes among others.

### Comparative genome analysis

**Fig. 2:**
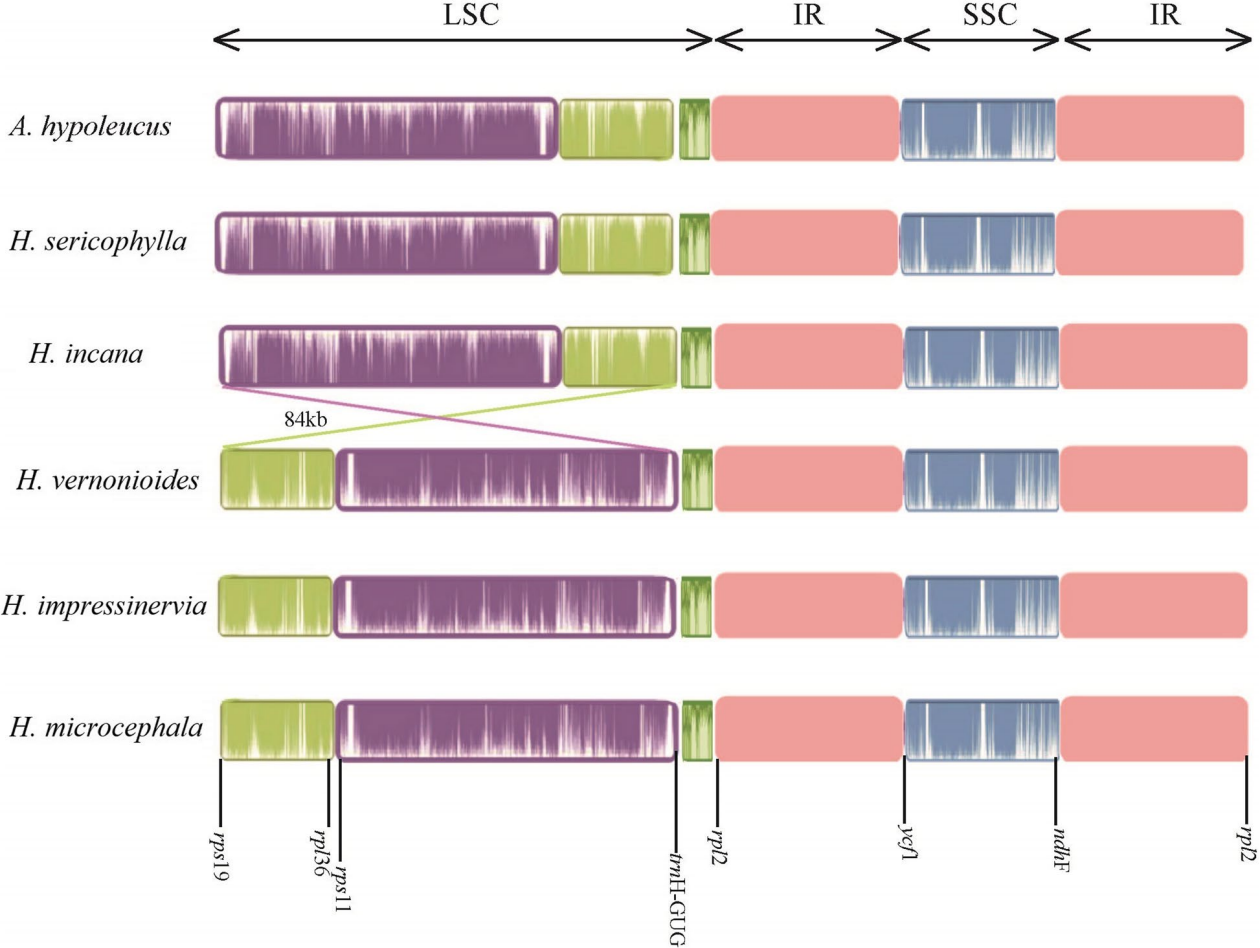
Plastid genome structure and gene order in the *Heteroplexis* chloroplast genomes compared with *Aster hypoleucus*

**Fig. 3:**
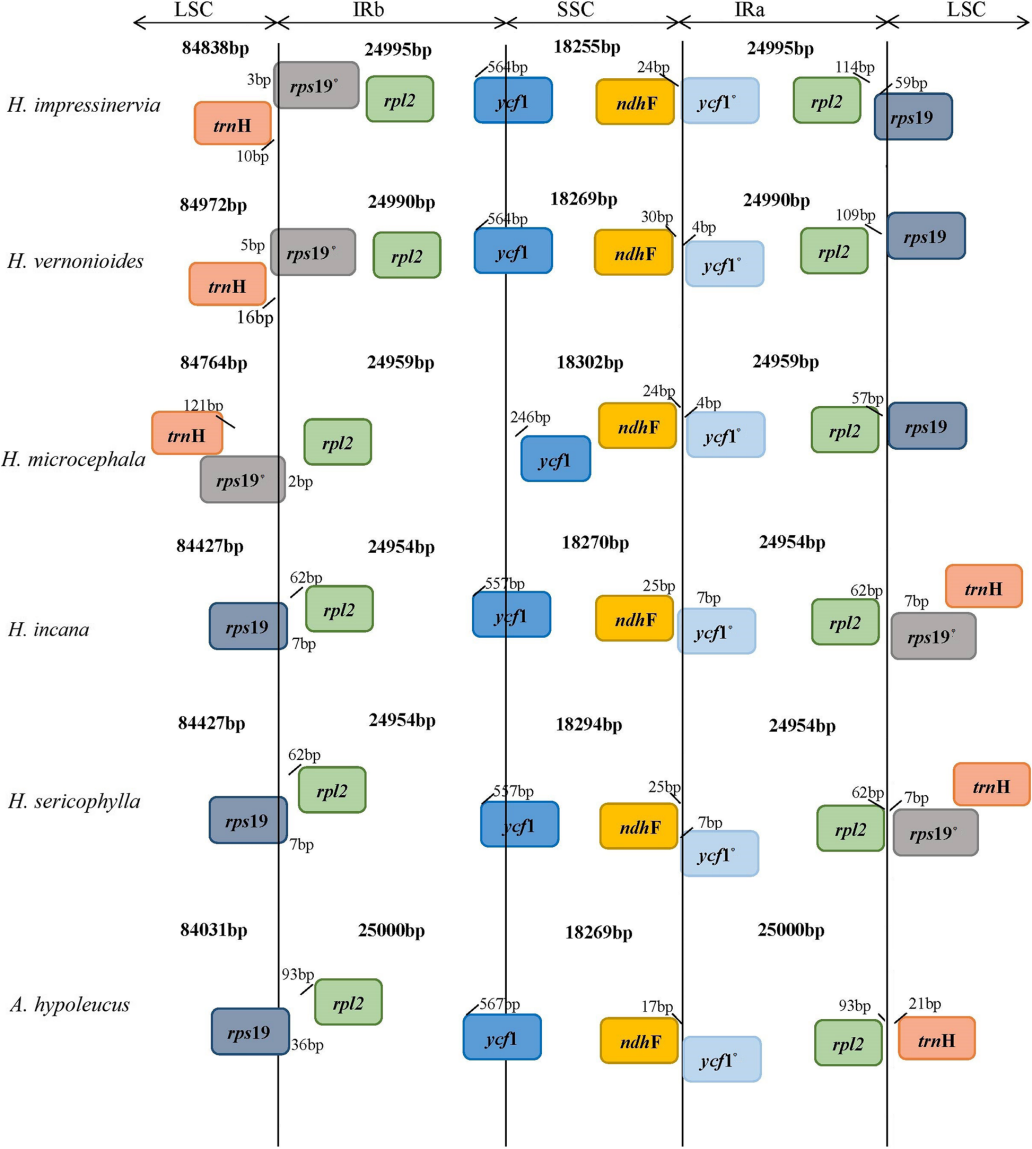
Comparison of the borders of LSC, SSC, and IR regions among chloroplast genomes of *Heteroplexis.* ^φ^ pseudogene

To understand the structural variation of *Heteroplexis* plastomes, we compared the cp. genomes of all five species and *A. hersileoides* with Mauve software. Our Mauve alignment (of the six cp. genomes) generated six locally collinear blocks (LCBs), each representing a homologous region without rearrangement (Fig. 2). The results revealed a large-scale inversion of the entire LSC region in *H. vernonioides*, *H. impressinervia* and *H. microcephala*, as shown in the altered gene positions at the two LSC/IR junctions (Fig. 3). Specific primers (Table S3) were developed to verify the authenticity of this inversion experimentally.

We compared the JL (LSC/IR) and JS (IR/SSC) boundary positions of the *Heteroplexis* plastomes (Fig. 3). The lengths of the IR regions were relatively uniform, ranging from 24,954 to 24,995 bp, with little contraction, especially for *H. impressinervia* and *H. vernonioides*. The JL (IR-LSC: *rpl*2 & *rps*19) boundary showed high similarity in five *Heteroplexis* plastomes, except for *H. impressinervia* and *H. vernonioides*. The *rps*19 gene crossed over the IR-LSC border and extended into the IR regions ranging for approximately 62 bp, resulting in a pseudo-copy *rps*19^φ^ (62 bp in length) by the duplication of IR regions. This pseudogene *rps*19^φ^ was jumped from JLB (IRb-LSC) to JLA (IRa-LSC) due to LSC region inversion in *H. impressinervia*, *H. vernonioides* and *H. microcephala*. In addition, the pseudogene *rps*19 was relocated the within the IRb region due to contraction of IR region, whereas the JLB (IRb-LSC: *rps*19 and *rpl*2) boundary genes were changed to *trn*H and *rps*19 in *H. impressinervia* and *H. vernonioides*. The JS (IR-SSC: *ycf*1 and *ndh*F) boundaries were also highly similar in *Heteroplexis* plastomes. The *ycf*1 gene crossed the IRb-SSC border and extended into the IRb region at approximately 564 bp, except in *H. microcephala*. This gene, like *rps*19 gene, also has a pseudo-copy *ycf*1^φ^ (564 bp in length) due to the duplication of IR regions.

**Fig. 4:**
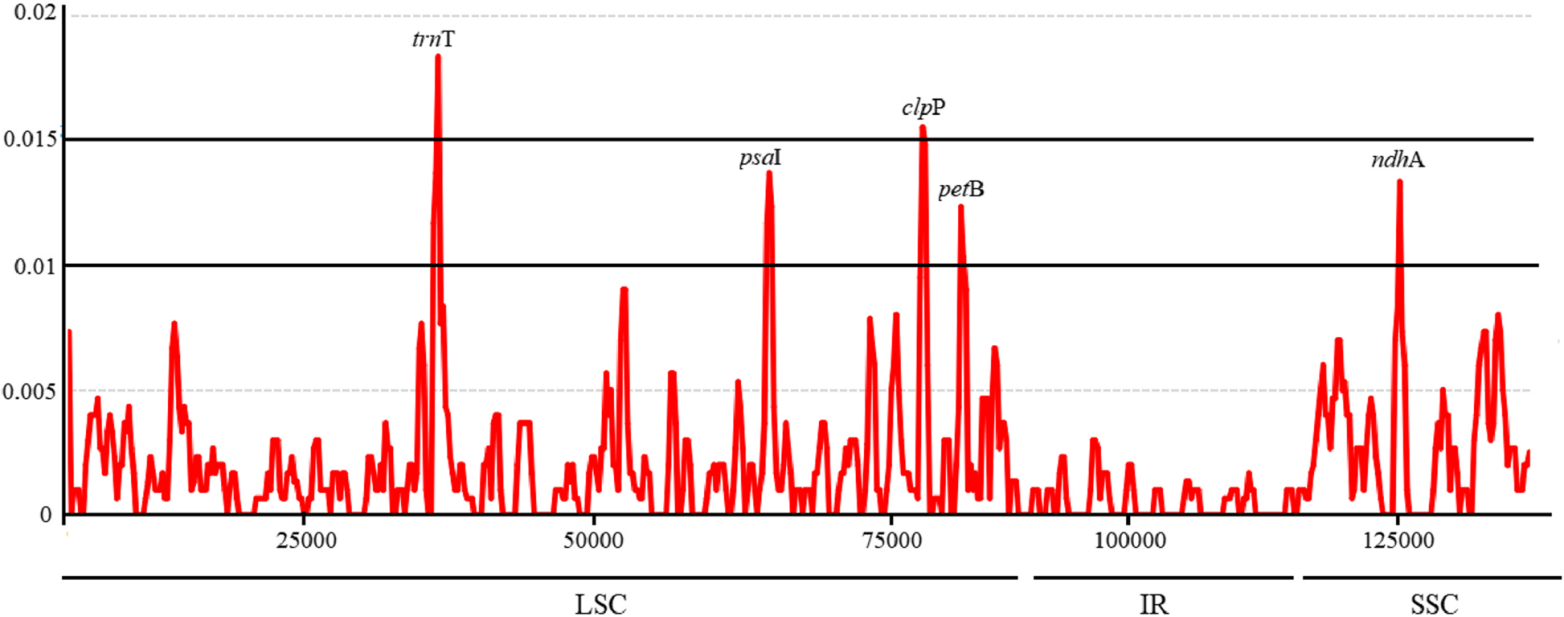
Nucleotide diversity in the *Heteroplexis* chloroplast genomes

The Pi (*π*) value in *Heteroplexis* plastomes ranged from 0 to 0.0183, with an average of 0.0017. The IR regions showed low nucleotide diversity, with most of the variations occurred in the LSC and SSC regions (Fig. 4). Although the protein-coding regions were conserved, five protein-coding genes (*trn*T, *psa*I, *clp*P, *pet*B and *ndh*A) showed significantly high *π* values (of > 0.01), with *trn*T gene showing the highest divergence value (0.0183) (Fig. 4). These polymorphic loci are candidate barcode sequences for phylogenetic inference and population genetic studies of the genus *Heteroplexis*.

### Phylogenetic analysis

**Fig. 5:**
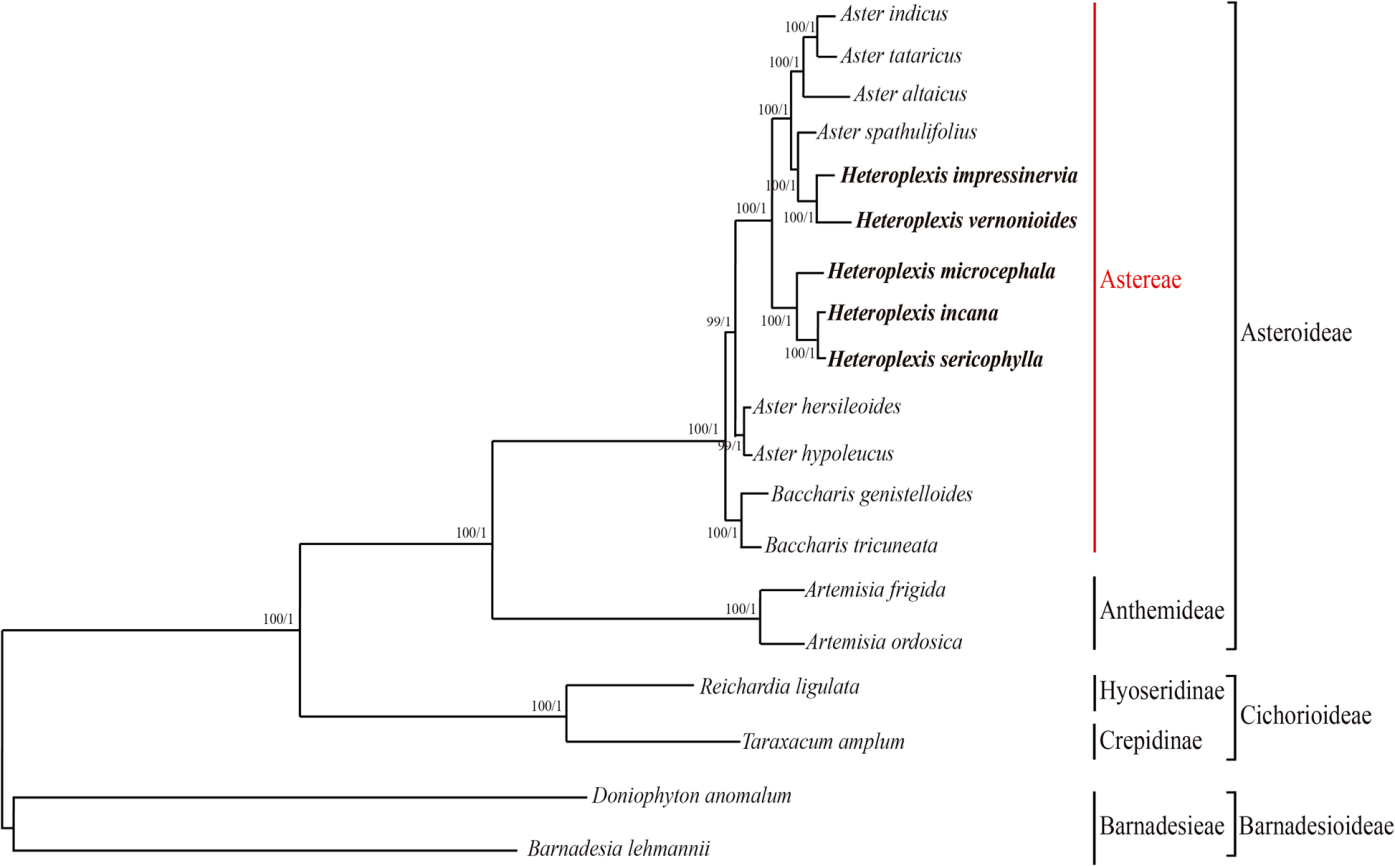
Maximum likelihood tree and Bayesian tree were constructed based on CDS data partitions of 19 species chloroplast genomes. The number on the branches as Bayesian inference posterior probability/maximum likelihood bootstrap support values

*Heteroplexis* genus consensus phylogeny was inferred from five data matrices (PCGs, LSC, SSC, IR and intergenic regions sequences) applying the best fit model (GTR + G) distance estimate under two different reconstruction methods (ML and BI), generating the same topology (Fig. 5 and Supplementary Figure S2). The plastid phylogenomic analysis identified a strongly supported (BS = 100, and PP = 1.0) phylogeny with two distinct not clustered clades: one harboring *H. vernonioides* and *H. impressinervia*, and the other clade with *H. microcephala*, *H. sericophylla* and *H. incana* (Fig. 5). This result revealed that the genus *Heteroplexis* was not monophyletic and embedded within the genus *Aster*. The clade formed by *H. vernonioides* and *H. impressinervia* was a sister clade to *Aster spathulifolius*, which at the same time was sister clade to other three *Aster* species, split from the second *Heteroplexis* cluster more basal to other *Aster* species. These results suggested that these five species should be subsumed into the genus *Aster*.

## Discussion

In this study, we reported three new plastomes of the genus *Heteroplexis* using Illumina sequencing and performed a series of genomic and phylogenetic analyses with previously published *Heteroplexis* plastomes. These new plastomes showed a typical quadripartite structure and their structure and gene content (113 unique genes) were highly conserved, as those species of the genus *Aster* [23]. Similarly to *Aster* species, 18 chloroplast protein-coding genes (six tRNA genes and 12 PCGs) were intron-containing genes [24], indicating the close relationship between the genus *Heteroplexis* and *Aster*.

The plastome of the most photosynthetic angiosperms is generally highly conserved in gene arrangement [17, 25]. However, plastome rearrangement is more common in the Compositae family. Except for Barnadesioideae, most clades have two inversions, a larger one of about 23 kb and a small one of about 3.3 kb, which may be a key feature in identifying species within the Compositae [26–28]. In *Heteroplexis*, a large-scale inversion of the entire LSC region was observed in *H. vernonioides*, *H. impressinervia* and *H. microcephala*. Although it is a common event that the cp. genome can exit in two orientations at the SSC region [29], the entire LSC region inversion is first found in *Heteroplexis* species.

The IR regions are an important part of the cp. genome, and its variation has been demonstrated to substantially contribute to the change in plastome size [30], and its duplication would also result in the pseudogenization of boundary genes [31]. The IR region sizes of the *Heteroplexis* plastomes were relatively stable, started around the *rps*19 gene, and terminated almost uniformly downstream of the *ycf*1 gene, as in *Aster* cp. genomes [23]. In particular, the *rps*19 and *ycf*1 genes shared the LSC/IRb and SSC/IRb boundaries, respectively. However, in the LSC/IRa and SSC/IRa boundaries, the genes were the pseudo-copy of these two parental genes. These results suggested that the duplication of the IR region resulted in the formation of partial copies of these genes, as in *Aster* species [23].

Previous studies of the genus *Heteroplexis* have consistently failed to resolve phylogenetic relationships, probably partly due to incomplete sampling or insufficient informative sites in the sampled sequences [23]. In contrast, we obtained well-supported clades, and all inter-specific relationships were well resolved (Fig. 5) by analysis of the whole plastome datasets, which was also supported by the whole genome-wide level restriction site-associated DNA sequencing (RADseq) data-sets [32]. Indicating that complete plastome data-sets have the potential to resolve the phylogenetic relationships of the genus, becoming guidance to resolve the actual taxonomic ambiguity of the *Heteroplexis* genus. Our strongly supported phylogeny, on which the *Heteroplexis* species are embedded within the genus *Aster*, is consistent with the results of an earlier study [7]. However, the split in two highly supported clusters (*H. vernonioides* and *H. impressinervia*; and *H. microcephala*, *H. sericophylla* and *H. incana*) corroborating the non-monophyly of *Heteroplexis* species differs from earlier studies [3, 7].

## Conclusion

By chloroplast genome comparative analysis in *Heteroplexis* species we determined that those plastomes, although highly conserved, had undergone extensive rearrangements, including gene losses, gene duplications, relocations, pseudogenization, IR contraction and LSC inversion. These new plastid data set provided new insights into the inter-specific relationships of *Heteroplexis* named specimens, suggesting that the five species might be subsumed into the *Aster* genus and cancel the *Heteroplexis* genus. Our results have laid the foundation for future studies on the taxonomy, phylogeny, and evolutionary history of *Aster *genus.

## Methods

### Sampling, sequencing and annotation

All five *Heteroplexis* species were included in this study, among which three species were investigated for the first time, namely *H. impressinervia* Chang, *H. vernonioides* Chang and *H. microcephala* Y. L. Chen; the voucher specimen storage information is shown in Supplementary Table S4. All samples were identified by Prof. Yancai Shi. The sampling of three newly sequenced species was approved by the Guangxi province of China and met local policy requirements. Our experimental research, including the collection of plant materials, complies with institutional, national, or international guidelines.

The fresh leaves were obtained from the field for DNA extraction. Total genomic DNAs were extracted using the CTAB method [33] and checked by visualization after 1% agarose-gel electrophoresis. The DNA was used to construct 150 bp insert libraries per the manufacturer’s manual (Illumina Inc., San Diego, CA, USA). The libraries were then subjected to sequencing. The high-throughput sequencing was performed on the Illumina HiSeq Platform (Illumina, San Diego, CA) at Genewiz in Suzhou, China. The Illumina paired-end data were cleaned with trimmomatic version 0.36 [34] and then used for cp. genome de novo assembly by the program NOVOPlasty version 4.3 [35]. Plastome annotation was performed using Plann version 1.1 [36] and the draft annotation was inspected and corrected manually in Sequin version 16.0 (http://www.ncbi.nlm.nih.gov/) [37]. The identities of all tRNA genes were further confirmed with the tRNA-SE search server version 1.4 [38]. A physical map of the genome was generated by OGDRAW version 1.3.1 (http://ogdraw.mpimp-golm.mpg.de/) [39].

### Repeat sequence analysis

Simple-sequence repeats were analyzed using MIcroSAtellite version 2.1 (MISA, https://webblast.ipk-gatersleben.de/misa) [40], with parameters set to 10, 5, 4, 3, 3, and 3 for mono-, di-, tri-, tetra-, penta-, and hexa-nucleotides respectively. The long repetitive sequences containing forward, palindromic, reverse, and complementary repeats were analyzed using the software REPuter version 1.1 (https://bibiserv.techfak.uni-bielefeld.de/reputer) [41] with a 30 bp minimum repeat size and a Hamming distance of 3. Tandem Repeats Finder version 4.04 (http://tandem.bu.edu/trf/trf.html) [42] was used to detect tandem repeats, with parameters set to 2 for the alignment parameter match and 7 for mismatches and indels.

### Comparative analyses and identification of highly divergent regions

All five *Heteroplexis* plastomes of *H. impressinervia* (MN367917), *H. vernonioides* (MN462631), *H. microcephala* (MW795355), *H. sericophylla* (MK942054) and *H. incana* (MN172194) were included in a comparative analysis. Firstly, the general features (including the genome structure, size, GC content, and gene content) of the five *Heteroplexis* cp. genomes were characterized using Geneious version 10.2.6 [43]. Secondly, Gene arrangements were further analyzed using Mauve alignment version 1.2 [44] with default parameters. The plastome of *Aster hersileoides* (NC_042944), which has a typical Astereae tribe plastome organization, was used as the reference in Mauve alignments. Thirdly, the junction of the plastomes was analyzed with IRscope version 3.1 (https://irscope.shinyapps.io/irapp/) [45]. Lastly, to further identify the hypervariable regions and the nucleotide diversity Pi (π), all five *Heteroplexis* chloroplast genome sequences were aligned using the MAFFT version 7.450 [46] with default parameters. The Pi (π) values were calculated using sliding window analysis (window length = 600 bp and step size = 200 bp excluding sites with alignment gaps) to detect highly divergent regions (i.e., mutation hotspots) in DnaSP version 6.0 [47].

### Phylogenetic analysis

To reconstruct the phylogenetic relationships, we complemented our data set of three *Heteroplexis* plastomes with two previously published *Heteroplexis* plastomes and 14 plastomes from Compositae (Supplementary Table S5), which included ten species from the Asteroideae subfamily, two species from Cichorioideae subfamily and two species from Barnadesioideae subfamily. The species *Barnadesia lehmannii* (MH341582) and *Doniophyton anomalum* (NC_048450) from the Barnadesioideae subfamily were used as an outgroup in the phylogenetic inference. We aligned the plastome sequences of all 19 selected species using MAFFT version 7.450 [46], with manual inspection and correction. We extracted three datasets from the finally aligned plastome matrix to assess the consistency of phylogenetic reconstructions based on different plastome regions. These included sequences of: (a) the protein-coding genes (PCGs), (b) LSC regions, (c) SSC regions, (d) IR regions, and (e) intergenic regions. We used maximum likelihood (ML) and Bayesian inference (BI) methods for phylogenetic analyses. Model Finder version 2.4 [48] was used to find the best model. The best-scoring ML tree was inferred using PhyML version 3.0 [49] with the GTRGAMMAX model for each partition, and clade support was assessed using the rapid bootstrap algorithm with 1,000 replicates. Four parallel Markov chain Monte Carlo (MCMC) runs were performed for the BI method using MrBayes version 3.1.2 [49, 50]. A total of 1,000,000 generations were run with sampling every 500 generations, and the first 25% of samples were discarded as burn-in. The consensus trees were finally edited using Figtree version 1.4.4 [51].

## Supplementary Information

**Additional file 1: Supplementary Material 1.**

**Additional file 2: Supplementary Material 2.**

## Data Availability

All annotated chloroplast genomes have been deposited in GenBank (https://www.ncbi.nlm.nih.gov/genbank/), accession numbers are provided in Supplementary Table S5.

## Abbreviations

IR: Inverted repeat
LSC: Large single copy
SSC: Small single copy
rRNA: Ribosomal rNA
tRNA: Transfer rNA
CTAB: Cetyltrimethylammonium bromide
PCR: Polymerase chain reaction
SSR: Simple sequence repeat
BI: Bayesian inference
ML: Maximum likelihood
BS: Branch support
