# Supplementary material for: Comparative and phylogenetic analysis based on chloroplast genome of Heteroplexis (Compositae), a protected rare genus
Source: BMC Plant Biol. 2022 Dec 22;22:605. doi: 10.1186/s12870-022-04000-1 (PMC9773445; doi:10.1186/s12870-022-04000-1)
Supplement: Supplementary file 1 — Additional file 1: Supplementary Material 1. [file 12870_2022_4000_MOESM1_ESM.doc]

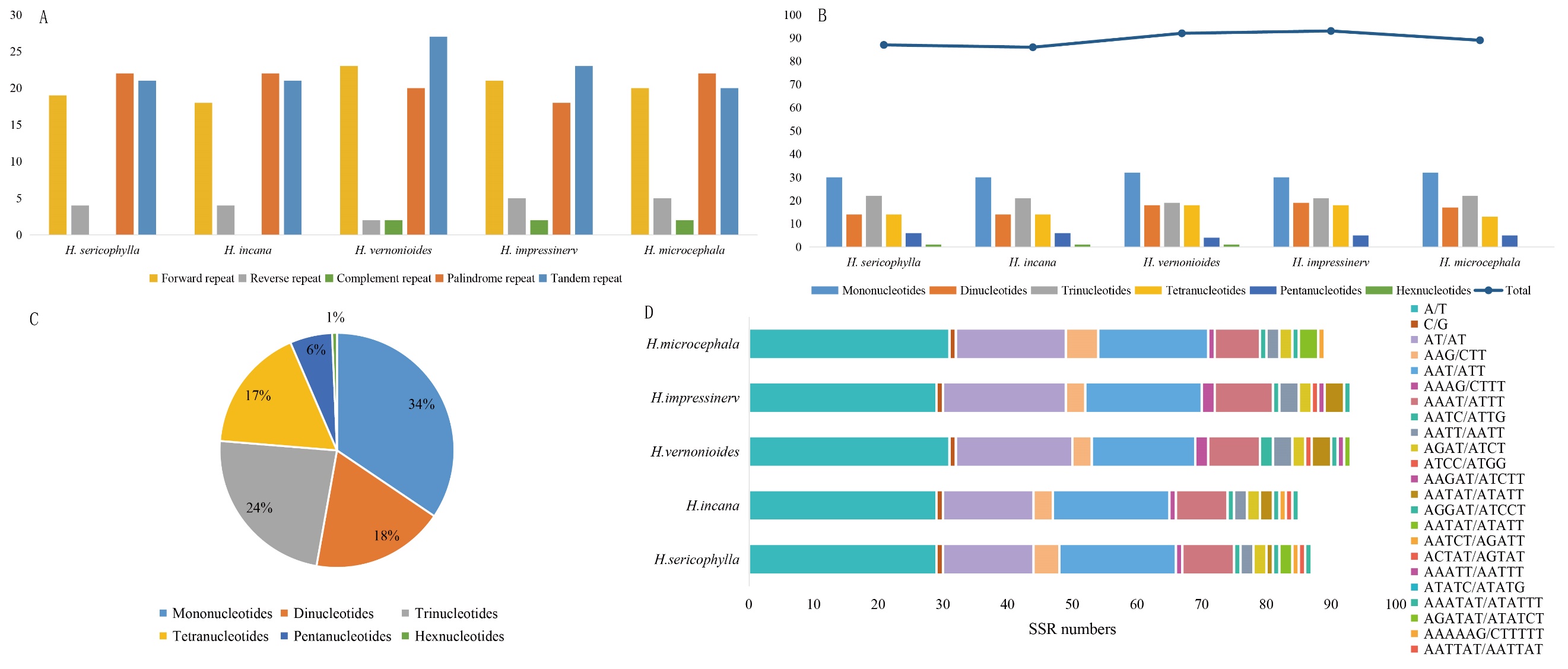


**Figure S1.** Quantitative analysis of various repeat types in *Heteroplexis* chloroplast genomes. (A) The number of Dispersed repeat and Tandem repeat (F: positive; R: reverse; C: Complement; P: Palindromic; T: tandem); (B) Number of various repeat types; (C) The proportion of repeat types in each species; (D) Number of SSRs.


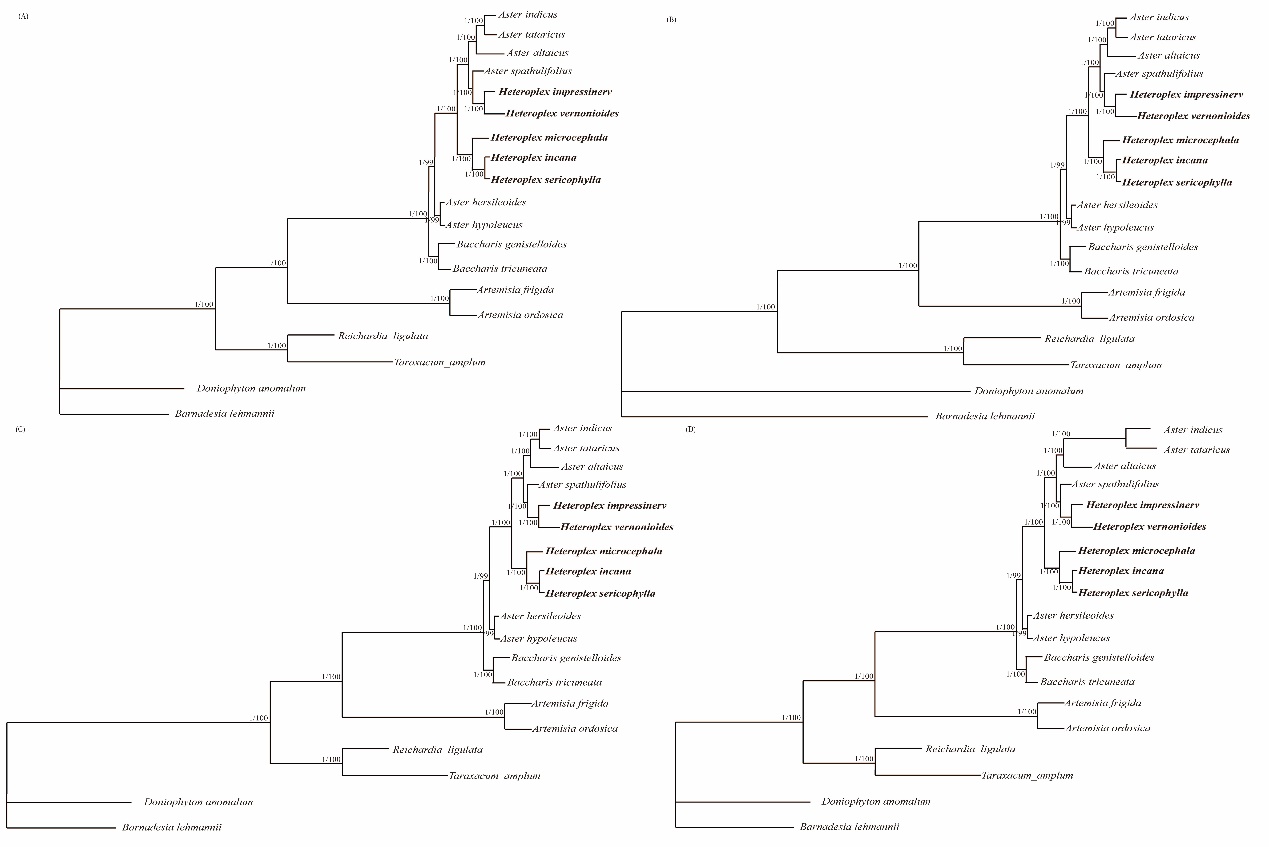


**Figure S2.** Phylogenetic tree of chloroplast genomes of *Heteroplexis* based on different data partitions. Support values are shown for nodes as Bayesian inference posterior probability/maximum likelihood bootstrap. (A) LSC regions, (B) IR regions, (C)SSC regions, (D) intergenic regions.
