# Supplementary material for: Comparative and phylogenetic analysis based on chloroplast genome of Heteroplexis (Compositae), a protected rare genus
Source: BMC Plant Biol. 2022 Dec 22;22:605. doi: 10.1186/s12870-022-04000-1 (PMC9773445; doi:10.1186/s12870-022-04000-1)
Supplement: Supplementary file 2 — Additional file 2: Supplementary Material 2. [file 12870_2022_4000_MOESM2_ESM.doc]

Table S1 Summary of characteristics of *Heteroplexis* chloroplast genomes.

| Spcies | 1. ***microcephala*** | ***H. impressinervia*** | ***H. vernonioides*** | *H. incana* | *H. sericophylla* |
| --- | --- | --- | --- | --- | --- |
| size(bp) | **152,984** | **153,083** | **153,221** | 152,605 | 152,629 |
| LSC(bp) | **84,764** | **84,838** | **84,972** | 84,427 | 84,427 |
| SSC(bp) | **18,302** | **18,255** | **18,269** | 18,270 | 18,394 |
| IRs(bp) | **24,959** | **24,995** | **24,990** | 24,954 | 24,954 |
| Total genes | **132** | **132** | **132** | 132 | 132 |
| Protein-coding genes | **85** | **85** | **85** | 85 | 85 |
| tRNA genes | **37** | **37** | **37** | 37 | 37 |
| rRNA genes | **8** | **8** | **8** | 8 | 8 |
| GC content | **37.30%** | **37.30%** | **37.20%** | 37.30% | 37.30% |
| Protein-coding% | **50.80%** | **49.50%** | **51.10%** | 51.40% | 51.30% |

Bold type indicates species sequenced in this study; data for others were downloaded from GenBank. LSC, large single copy; SSC, small single copy; IR, inverted repeat.

Table S2 List of annotated genes in Heteroplexis chloroplast genome.

| Category | Group | Name (s) |
| --- | --- | --- |
| Photosynthesis | NADPH dehydrogenase | *ndhA, *^a^ndhB, ndhC, ndhD, ndhE, ndhF,  ndhG, ndhH, ndhI, ndhJ, ndhK |
|  | Photosystem I | psaA, psaB, psaC, psaI, psaJ |
|  | Photosystem II | psbA, psbB, psbC, psbD, psbE, psbF, psbH, psbI,  psbJ, psbK, psbL, psbM, psbN, psbT, psbZ |
|  | ATP synthase | atpA, atpB, atpE, *atpF, atpH, atpI |
|  | cytochrome b/f complex | petA, *petB, *petD, petG, petL, petN |
|  | Rubisco | rbcL |
| Self-replication | transfer RNA | *^a^trnA-UGC, trnC-GCA, trnD-GUC, trnE-UUC, trnF-GAA,  trnfM-CAU, trnG-GCC, *trnG-UCC, trnH-GUG, ^a^trnI-CAU,  *^a^trnI-GAU, *trnK-UUU, ^a^trnL-CAA, *trnL-UAA, trnL-UAG,  trnM-CAU, ^a^trnN-GUU, trnP-UGG, trnQ-UUG, ^a^trnR-ACG,  trnR-UCU, trnS-GCU, trnS-GGA, trnS-UGA, trnT-GGU,  trnT-UGU, ^a^trnV-GAC, *trnV-UAC, trnW-CCA, trnY-GUA |
|  | ribosomal RNA | ^a^rrn4.5, ^a^rrn5, ^a^rrn16, ^a^rrn23 |
|  | RNA polymerase | rpoA, rpoB, *rpoC1, rpoC2 |
|  | Small subunit of ribosomal | rps2, rps3, rps4, ^a^rps7, rps8, rps11, **^a^rps12, rps14, rps15, *rps16, rps18, ^a φ^rps19 |
|  | Large subunit of ribosomal | *^a^rpl2, rpl14, *rpl16, rpl20, rpl22, ^a^rpl23, rpl32, rpl33, rpl36 |
| Other genes | translational initiation factor | infA |
|  | proteolysis | **clpP |
|  | Maturase | matK |
|  | Subunit of acetyl-CoA | accD |
|  | envelop membrane protein | cemA |
|  | c-type cytochrome synthesis gene | ccsA |
| Genes of unknown function | hypotetical chloroplast reading frames | ^a φ^ycf1, ^a^ycf2, **ycf3, ycf4 |
| * Indicates genes containing one or more introns, a gene with two copies, a φ indicates genes containing one pseudogene. | | |

Table S3 Primer pairs designed for verification.

| Name(s) | Sequences(5'to3') |
| --- | --- |
| H_vimc-IRaF | AGCGCCCTGTAGTAAGAGGA |
| H_vimc-IRaR | GGTGCGAATTCTCCTAATTTGTGT |
| H_vimc-IRbF | ACCGTGCTAACCTTGGTATG |
| H_vimc-IRbR | GGAGGAAGCTGTGACACG |
| H_si-IRaF | ATCCACATGGGGGTGGTGAA |
| H_si-IRaR | AAAGAGGGCGTTATTGCTCC |
| H_si-IRbF | GTGTCCTACCATACGATCCG |
| H_si-IRbR | CTAGGTAAGCGCCCTGTAGT |

Table S4 Sampling information for *Heteroplexis* species in this study.

| **Species** | **Locality** | **longitude** | **latitude** | **Elevation(m)** | **Voucher** | **Source** |
| --- | --- | --- | --- | --- | --- | --- |
| ***H. vernonioides*** | Liuzhou, Guangxi | 107°8′21″ | 107°8′21″ | 513 | liu20190617_ylj | Guangxi Institute of Botany |
| ***H. microcephala*** | Guilin, Guangxi | 110°34′2″ | 24°51′22″ | 172 | liu20190617_xh | Guangxi Institute of Botany |
| ***H. impressinervia*** | Chongzuo, Guangxi | 106°55′26″ | 22°36′2″ | 560 | liu20190617_am | Guangxi Institute of Botany |

Table S5 List of species used for phylogenetic tree construction.

| Family | Subfamily | Tribe | Genus | Spcies | GeneBank accession |
| --- | --- | --- | --- | --- | --- |
| Compositae | Asteroideae | Astereae | *Heteroplexis* | *Heteroplexis impressinervia* | MN367917 |
| Compositae | Asteroideae | Astereae | *Heteroplexis* | *Heteroplexis microcephala* | MW795355 |
| Compositae | Asteroideae | Astereae | *Heteroplexis* | *Heteroplexis incana* | MN172194 |
| Compositae | Asteroideae | Astereae | *Heteroplexis* | *Heteroplexis sericophylla* | MK942054 |
| Compositae | Asteroideae | Astereae | *Heteroplexis* | *Heteroplexis vernonioides* | MN462631 |
| Compositae | Asteroideae | Astereae | *Aster* | *Aster spathulifolius* | NC_027434 |
| Compositae | Asteroideae | Astereae | *Aster* | *Aster indicus* | NC_040126 |
| Compositae | Asteroideae | Astereae | *Aster* | *Aster hersileoides* | NC_042944 |
| Compositae | Asteroideae | Astereae | *Aster* | *Aster hypoleucus* | NC_046503 |
| Compositae | Asteroideae | Astereae | *Aster* | *Aster tataricus* | NC_042913 |
| Compositae | Asteroideae | Astereae | *Aster* | *Aster altaicus* | KX352465 |
| Compositae | Asteroideae | Astereae | *Baccharis* | *Baccharis tricuneata* | NC_034868 |
| Compositae | Asteroideae | Astereae | *Baccharis* | *Baccharis genistelloides* | NC_034852 |
| Compositae | Asteroideae | Anthemideae | *Artemisia* | *Artemisia frigida* | NC_020607 |
| Compositae | Asteroideae | Anthemideae | *Artemisia* | *Artemisia ordosica* | NC_046571 |
| Compositae | Cichorioideae | Hyoseridinae | *Reichardia* | *Reichardia ligulata* | MN893255 |
| Compositae | Cichorioideae | Crepidinae | *Taraxacum* | *Taraxacum amplum* | NC_031816 |
| Compositae | Barnadesioideae | Barnadesieae | *Barnadesia* | *Barnadesia lehmannii* | MH341582 |
| Compositae | Barnadesioideae | Barnadesieae | *Doniophyton* | *Doniophyton anomalum* | NC_048450 |
